# Protocol for a cluster randomised controlled feasibility study of Prehospital Optimal Shock Energy for Defibrillation (POSED)

**DOI:** 10.1016/j.resplu.2022.100310

**Published:** 2022-10-06

**Authors:** Helen Pocock, Charles D. Deakin, Ranjit Lall, Felix Michelet, Abraham Contreras, Mark Ainsworth-Smith, Phil King, Anne Devrell, Debra E. Smith, Gavin D. Perkins

**Affiliations:** a Warwick Clinical Trials Unit, Warwick Medical School, University of Warwick, Gibbet Hill Road, Coventry CV4 7AL, United Kingdom; b South Central Ambulance Service NHS Foundation Trust, Southern House, Sparrowgrove, Otterbourne, Winchester, SO21 2RU, United Kingdom; c University Hospital Southampton NHS Foundation Trust, Tremona Road, Southampton SO16 6YD, United Kingdom; d University Hospitals Birmingham NHS Foundation Trust, Birmingham Heartlands Hospital, Bordesley Green East, Birmingham B9 5SS, United Kingdom; e PPI Representative, Warwick Clinical Trials Unit, Warwick Medical School, University of Warwick, Gibbet Hill Road, Coventry, CV4 7AL, United Kingdom

**Keywords:** Defibrillation, Out-of-Hospital Cardiac Arrest, Ventricular Fibrillation, Electric Countershock, Cardiopulmonary Resuscitation, Feasibility study, AE, Adverse Event, AOR, Adjusted Odds Ratio, B-CPR, Bystander CPR, BTE, Biphasic Truncated Exponential waveform, CAD, Computer Aided Despatch, CONSORT, CONsolidated Standards Of Reporting Trials, CPMS, Central Portfolio Management System, CPR, Cardiopulmonary Resuscitation, CRF, Case Report Form, ePR, Electronic Patient Record, GCP, Good Clinical Practice, HRA, Health Research Authority, ICA, Integrated Clinical and practitioner Academic programme, ILCOR, International Liaison Committee on Resuscitation, ISRCTN, International Standard Registered Clinical/social sTudy Number, J, Joules, JRCALC, Joint Royal Colleges Ambulance Liaison Committee, mRS, Modified Rankin Scale, NIHR, National Institute for Health and care Research, OHCA, Out-of-Hospital Cardiac Arrest, OR, Odds Ratio, PEA, Pulseless Electrical Activity, POSED, Prehospital Optimal Shock Energy for Defibrillation, PPI, Patient and Public Involvement, pVT, Pulseless Ventricular Tachycardia, REC, Research Ethics Committee, RFA, Rankin Focused Assessment, ROSC, Return of Spontaneous Circulation, ROOR, Return of Organised Rhythm, SMG, Study Management Group, SOC, Study Oversight Committee, SPIRIT, Standard Protocol Items: Recommendations for Intervention Trials, ToF, Termination of Fibrillation, VF, Ventricular Fibrillation, WCTU, Warwick Clinical Trials Unit

## Abstract

**Aims:**

The Prehospital Optimal Shock Energy for Defibrillation (POSED) study will assess the feasibility of conducting a cluster randomised controlled study of clinical effectiveness in UK ambulance services to identify the optimal shock energy for defibrillation.

**Methods:**

POSED is a pragmatic, allocation concealed, open label, cluster randomised, controlled feasibility study. Defibrillators within a single UK ambulance service will be randomised in an equal ratio to deliver one of three shock strategies 120–150–200 J, 150–200–200 J, 200–200–200 J. Consecutive adults (≥18 years) presenting with out of hospital cardiac arrest requiring defibrillation will be eligible. The study plans to enrol 90 patients (30 in each group). Patients (or their relatives for non-survivors) will be informed about trial participation after the initial emergency has resolved. Survivors will be invited to consent to participate in follow-up (i.e., at 30 days or discharge).

The primary feasibility outcome is the proportion of eligible patients who receive the randomised study intervention. Secondary feasibility outcomes will include recruitment rate, adherence to allocated treatment and data completeness. Clinical outcomes will include Return of an Organised Rhythm (ROOR) at 2 minutes post-shock, refibrillation rate, Return of Spontaneous Circulation (ROSC) at hospital handover, survival and neurological outcome at 30 days**.**

**Conclusion:**

The POSED study will assess the feasibility of a large-scale trial and explore opportunities to optimise the trial protocol.

**Trial registration:** ISRCTN16327029.

## Highlights

• Evidence for best shock energy to treat out-of-hospital cardiac arrest is lacking.
• POSED is a feasibility study for a future trial intended to close the evidence gap.
• 90 patients will be recruited within a single NHS Ambulance Service.
• Feasibility will be tested by comparing numbers of eligible with recruited patients.
• We will select best outcomes for a trial based on clinical relevance, reliability and ease of recording.

## Introduction

### Background and rationale

Globally between 30 and 97 patients per 100,000 population receive treatment by Emergency Medical Services for cardiac arrest each year.^1^ Defibrillation is the mainstay treatment for patients displaying ventricular fibrillation or pulseless ventricular tachycardia, but even following a witnessed collapse, only 10–33% survive with favourable neurological outcome.^1^ Chances of survival decrease with increasing duration of ventricular fibrillation.^2^ Amongst witnessed cases, the chance of survival to 30 days decreases with each shock (odds ratio (OR) 0.9; 95% Confidence Interval (CI) 0.88–0.92).^3^ An optimal shock energy strategy can shorten the resuscitation duration. This is important as longer resuscitation durations (31 minutes versus 5 minutes) are associated with a significantly lower chance of survival with a favourable neurological outcome (adjusted OR 0.04; 95% CI 0.03–0.05).^4^

### First shock success

Although several studies report first shock success, none has provided sufficient strength of evidence on which to base treatment recommendations. As such, current guidelines permit the use of any defibrillation energy level between 120–360 J.^5^ The International Liaison Committee on Resuscitation (ILCOR) has identified defibrillation energy levels as a priority area for research.^6^

A previous systematic review reported no observed difference in first shock success for energy levels between 120 and 200 J.^7^ Eight identified papers reported data from six studies but these compared a variety of waveforms, different measures of shock success and were undertaken at a time when resuscitation protocols recommended 3 stacked shocks and less focus on high-quality CPR. Of studies using 2010 CPR guidelines, with the emphasis on early defibrillation and single shocks separated by high-quality CPR, two report first shock success.^8^, ^9^ The first of these compares manual versus automated delivery of 360 J biphasic truncated exponential (BTE) waveform shocks, where fibrillation was terminated in 80.7–84.3% of cases.^8^ The second paper does not report energies and includes two different biphasic waveforms, but does not distinguish between them when reporting outcomes.^9^

### Shock strategy

Should further shocks be required, no strong evidence favours delivery at the same level or escalating the energy.^10^ In the BIPHASIC trial, escalating high-energy protocol was more successful than fixed low-energy protocol at both terminating fibrillation (81.8% vs 71.8%) and restoring an organised rhythm (36% vs 25.7%).^11^ However, a stacked shocks regime was employed, and the study was underpowered to detect a difference in survival. Our recent systematic review identified a recent post-hoc analysis of the CIRC trial, comparing manual and mechanical chest compression delivery, which explored the effect of shock strategy.^12^ No difference in long-term survival was detected between patients receiving escalating energy shocks (200–300–360 J) and those receiving fixed high-energy shocks (360–360–360 J) (unadjusted risk ratio 0.99, 95% CI 0.73, 1.23).^13^ However, no strong conclusions can be drawn due to the post-hoc non-randomised nature of the analysis.

There is a clear need for further research to determine the optimal shock energy strategies for first and subsequent shocks, and their effects on clinical outcomes. However, whether such a trial is feasible is currently unknown. Therefore, this study aims to determine: *Is it feasible to conduct a randomised, pragmatic clinical effectiveness trial in UK ambulance services to identify the optimal energy for defibrillation?* The primary objective of this study is to establish whether it is feasible to conduct a large-scale definitive trial based on the number of eligible patients and the number recruited. The secondary objectives are (1) to measure the rate of adherence to the allocated treatment, (2) identify the best outcome measures in terms of clinical relevance, ease and reliability of recording. We present the study according to SPIRIT guidelines (Appendix A1).^14^

## Methods

### Protocol

The full protocol and statistical analysis plan and any future updates are available on the trial website: https://warwick.ac.uk/fac/sci/med/research/ctu/trials/posed/.

The protocol was developed in accordance with the principles of the Declaration of Helsinki and Good Clinical Practice (GCP) and guidance from the Standard Protocol Items: Recommendations for Intervention Trials (SPIRIT).^14^ Core study information is presented in Appendix A2. The study is conducted and managed by the Warwick Clinical Trials Unit (WCTU) and sponsored by University of Warwick, Research & Impact Services, University House, University of Warwick, Coventry, CV4 8UW.

### Study design

POSED is a pragmatic, allocation-concealed, open label, cluster randomised (by defibrillator) controlled feasibility study with a 1:1:1 allocation ratio.

### Study setting

Recruitment to this single-site study will be led by South Central Ambulance Service, an NHS ambulance service in the south of England. The trial opened to recruitment on 22 March 2022.

### Eligibility criteria

Patients will be included if they sustain an OHCA attended by a crew carrying a study defibrillator, resuscitation is attempted and a shock delivered. Patients known or suspected to be under 18 years old are excluded.

### Study interventions

Defibrillators are routinely pre-programmed to deliver consistent 1st, 2nd and subsequent shock energies within an ambulance service. These pre-programmed energies are the default settings whether used in automatic or manual mode. In this study, Zoll X-series defibrillators (rectilinear biphasic waveform) have been randomised to deliver one of three shock strategies as described in Table 1. Strategies are pre-programmed by the researcher to optimise protocol adherence and remain default settings in both automatic and manual mode. Protocol adherence will be monitored via the study database; when data from defibrillators is entered, any discrepancy between allocated and delivered energy will automatically raise an alert on the database.

**Table 1:** Energy levels of treatment groups (J = joules).

Group	First shock	Second shock	Subsequent shocks	Strategy	Intervention/Comparator
1	120 J	150 J	200 J	Escalating	Comparator
2	150 J	200 J	200 J	Escalating	Intervention
3	200 J	200 J	200 J	Fixed	Intervention

The treatment groups represent three variations in current UK practice. Group 1, the most commonly used strategy, delivers shock energies according to manufacturer’s current guidance. Group 2 and 3 interventions provide higher first shock energies, escalating or maintaining at the highest energy for the second and subsequent shocks.

The treatment allocation determines the energy levels of the first three sequential shocks. Should a return of spontaneous circulation (ROSC) be observed and then refibrillation occur, the patient will receive the next sequential allocated energy level. If the defibrillator is changed during a patient care episode, normal site policy will apply regarding shock energy escalation (i.e. second and subsequent shocks to be delivered at 200 J).

Clinical management of patients will adhere to the 2021 Resuscitation Council UK Advanced Life Support Guidelines.^5^ The study protocol does not mandate or prohibit any specific concomitant care. Study treatment ceases either when out-of-hospital resuscitation efforts are discontinued or upon handover of care to the hospital team who will deliver their usual care.

### Outcome measures

#### Primary outcome measure

The primary feasibility outcome is the proportion of eligible patients who receive the randomised intervention.

#### Secondary outcome measures

The secondary feasibility outcomes are**:**

• Treatment adherence rate.
• Data completeness of the following clinical outcomes:
  o Return Of Organised Rhythm (ROOR) 2 min post shock
  o Resulting rhythm (VF/pulseless VT/pulseless electrical activity (PEA)/asystole) 2 min post shock
  o Re-arrest rate (re-fibrillation)
  o Survived event (ROSC at hospital handover)
  o Survived to 30 days and hospital discharge
  o Neurological function (mRS score) at 30 days and hospital discharge

In addition to the outcomes described above, the study will also assess the acceptability of approach to informing relatives of non-survivors.

We will also report process outcomes including CPR metrics and resuscitation treatments (see Appendix A3). A schedule of assessments is provided in Table 2.

**Table 2:** Study outcome assessment time points, POSED, Prehospital Optimal Shock Energy for Defibrillation.

	Cardiac arrest	Hospital	Day 30
Inclusion/exclusion criteria	✓	✗	✗
Cardiac arrest data	✓	✗	✗
Patient identifiers	✓	✓	✗
Adverse event reportingo	✓	✓	✗
National data opt-out check	✓		✗
Survival checks	✓	✓	✗
Survival status	✓	✓	✓
Hospital stay data	✗	✓	✗
Notification of enrolment and invitation to take part in follow up	✗	✓	✗
Informed consent	✗	✓	✗
Neurological outcome (mRS)	✗	✗	

### Patient timeline

Participants are enrolled on receipt of the randomised intervention (i.e., when a shock from a randomised defibrillator is delivered to an adult patient). A time schedule is provided in Appendix A4.

### Sample size

This feasibility study aims to recruit 90 patients, ideally 30 in each arm. This is in keeping with a recommended sample size of at least 50 for feasibility studies,^15^ whilst allowing roughly equal recruitment into each arm. In order to reach target sample size, defibrillators on every vehicle based in the study area (*n* = 36) were randomised and pre-programmed, eliminating the additional task of selecting the intervention energy at the patient’s side.

### Allocation

#### Sequence generation

The unit of randomisation is the defibrillator (cluster), which is allocated to one of the three treatment groups in a 1:1:1 ratio using simple randomisation, stratified by vehicle type (ambulance, response car, team leader vehicle). Ambulance staff are allocated to appropriate vehicles by rota, so in practice will use defibrillators randomised across all three strategies.

#### Allocation concealment mechanism

Randomisation was achieved using the callsigns of the vehicles carrying the defibrillators. The callsign was allocated to one of the treatment groups (1, 2 or 3) ensuring allocation concealment from the ambulance crews.

#### Implementation

The allocation sequence was computer-generated, and the allocations programmed into the defibrillators by the researcher.

### Blinding

At the point of defibrillator charging, ambulance staff will see the pre-set treatment allocation and so will not be blinded. The clinical outcomes assessed following randomisation are largely objective in nature, so bias due to unblinding should not be an issue. However, we will monitor the data to assess any peculiarities which may be suspicious in the data. Due to the clinical nature of cardiac arrest, patients will be blind to treatment allocation. Control room vehicle dispatchers will be unaware of the treatment allocation. Hospital staff treating patients may be blind to the patient’s treatment allocation since the shock energy is not routinely included in the verbal handover. If patients ask what treatment they received, they will be asked not to pass this information to the research paramedic as the modified Rankin Scale (mRS) assessment should be completed blind to the treatment allocation.

### Data collection methods

Eligible patients are identified from Ambulance service Computer Aided Despatch (CAD) data and Electronic Patient Records (ePR). Data downloaded from defibrillators will indicate the treatment received.

Enrolments will be identified by research paramedics via ambulance service electronic systems reports. We will extract patients’ out-of-hospital data and ambulance response data from routinely collected ambulance service records. Patient and event characteristics, Utstein variables, out-of-hospital treatments and initial outcome will be sought from the patient clinical record. Defibrillator data (treatment summary report) can be downloaded from Zoll X-series devices onto USB devices and viewed via Zoll ‘RescueNet™ Code Review’ software (v5.9.0.5).^16^ Rhythm interpretation will be performed by HP and checked by CDD. We will assess mRS using the Rankin Focussed Assessment (RFA) tool which tool has excellent inter-observer reliability (unweighted κ of 0.93 (95% CI, 0.85–1.00)).^17^

Study data shall be entered at site onto electronic Case Report Forms (CRFs) on a secure university-hosted database, accessible only to authorised personnel.

Following enrolment, we will meet patients face to face in hospital where possible or will write to them following discharge if not. We prefer the former approach as it demonstrates the requisite sensitivity and respect and, in our previous experience, promotes participant retention. We will continue to collect and analyse patient data for those in receipt of an intervention which deviated from the protocol. Site shall perform Critical Data Item checks of dates of birth; should we find that a patient under 18 years old has been entered into the study, no further data shall be collected. In both cases, the non-compliance protocol shall be followed.

### Data management

No patient-identifiable information will be held by the university. This will be held by the ambulance service for the purpose of contact for information provision and follow-up. The university-hosted database shall include inbuilt validation checks. Site shall follow a single data entry process with a subsequent 10% check performed by a different team member. We will present recruitment and accumulating data on a monthly basis to the Study Management Group (SMG) and a six-monthly basis to the Study Oversight Committee (SOC) for review.

### Statistical methods

We will present summary statistics, namely mean, standard deviation, median, interquartile ranges and missingness in the data. Where possible we will also provide 95% confidence intervals.

Baseline and process data will be presented using descriptive statistics; categorical variables will be presented as proportions and continuous variables as medians with ranges stated. We shall report the proportion of patients in each arm (intention-to-treat) with complete data. No formal statistical comparisons of outcomes will be performed as the study is not powered to detect differences between the interventions.

We will report treatment adherence rate in terms of how many patients received the allocated first shock energy and, where applicable, how many received the correct subsequent shock energies. Additionally, we shall conduct sub-group analyses of survival status at hospital handover, discharge and 30 days where the initial rhythm is shockable (VF/pVT) to explore any interaction between sub-groups and treatment arm. Full details can be found in the Statistical Analysis Plan on the study website.

We will assess the feasibility of informing the relatives of non-survivors by recording (1) the proportion of patients where contact details for a relative was identified, (including time taken to identify relatives, source of information and concordance/discordance between sources) (2) proportion of sent letters returned as unknown at that address (3) record the number and nature of enquiries received by the Ambulance service in response to receipt of a letter.

### Data monitoring

A Study Oversight Committee has been appointed, led by an independent chair, comprising the co-chief investigators, study statistician and two lay representatives. There will be no Data Monitoring Committee since this feasibility study requires no interim analyses and data will be assessed for completeness rather than for efficacy/effectiveness. The Study Management Group, consisting of the project staff and co-investigators involved in the day-to-day running of the study, will meet monthly. Significant issues arising from management meetings will be referred to the Study Oversight Committee or Investigators, as appropriate. Throughout the project, we will continue to collaborate with the local Patient and Public Involvement (PPI) advisory panel.

### Harms

A safety reporting protocol has been developed for related and unexpected serious adverse events and directly attributable adverse events (AEs). An AE is defined as any untoward medical occurrence in a patient which does not necessarily have a causal relationship with the intervention. The trial Chief Investigator, with input from the WCTU Quality Assurance team, will determine whether AEs require reporting to the trial sponsor, SOC and Research Ethics Committee (REC), in accordance with the full safety reporting protocol. Adverse events (AEs) occurring within 30 days of treatment, can be reported using electronic AE forms, or via email.

### Auditing

The trial coordinating centre shall conduct remote monitoring halfway through the recruitment period.

### Ethics

The study protocol has been approved by the London (Harrow) Research Ethics Committee (20/LO/1242). The study is conducted in accordance with the Declaration of Helsinki, Good Clinical Practice (GCP) guidelines and the Mental Capacity Act 2005. We have engaged extensively with patients and public in considering the ethical issues inherent in this emergency research. Unfortunately, most patients sustaining out-of-hospital cardiac arrest will not survive. At this difficult time, any unnecessary emotional burden ought to be avoided but openness about the study may prevent further upset later. We have engaged extensively both with patient and public groups, and with researchers around the world in deciding how best to provide information.

### Consent

The process for obtaining consent in this study is being conducted in accordance with the Mental Capacity Act (2005) and has the approval of the Research Ethics Committee.

Since the study intervention will already have been given at this point, consent is sought only for follow-up. Research paramedics shall seek survivors’ consent when they have left the critical care area and been moved to a ward. The timing of this approach will vary between patients depending on the length of their critical care stay. Where survivors do not regain capacity a consultee (personal or nominated) will be sought to provide information about the patient’s enrolment and seek their opinion as to whether the patient would object to taking part in the follow-up phase of the study. Where patients do not survive, no follow up is conducted and so consent is not sought. A model consent form and Patient Information Sheet are presented in Appendix A5. In exceptional cases, if we are unable to make contact in hospital, we may arrange a home visit.

**Fig. 1:**
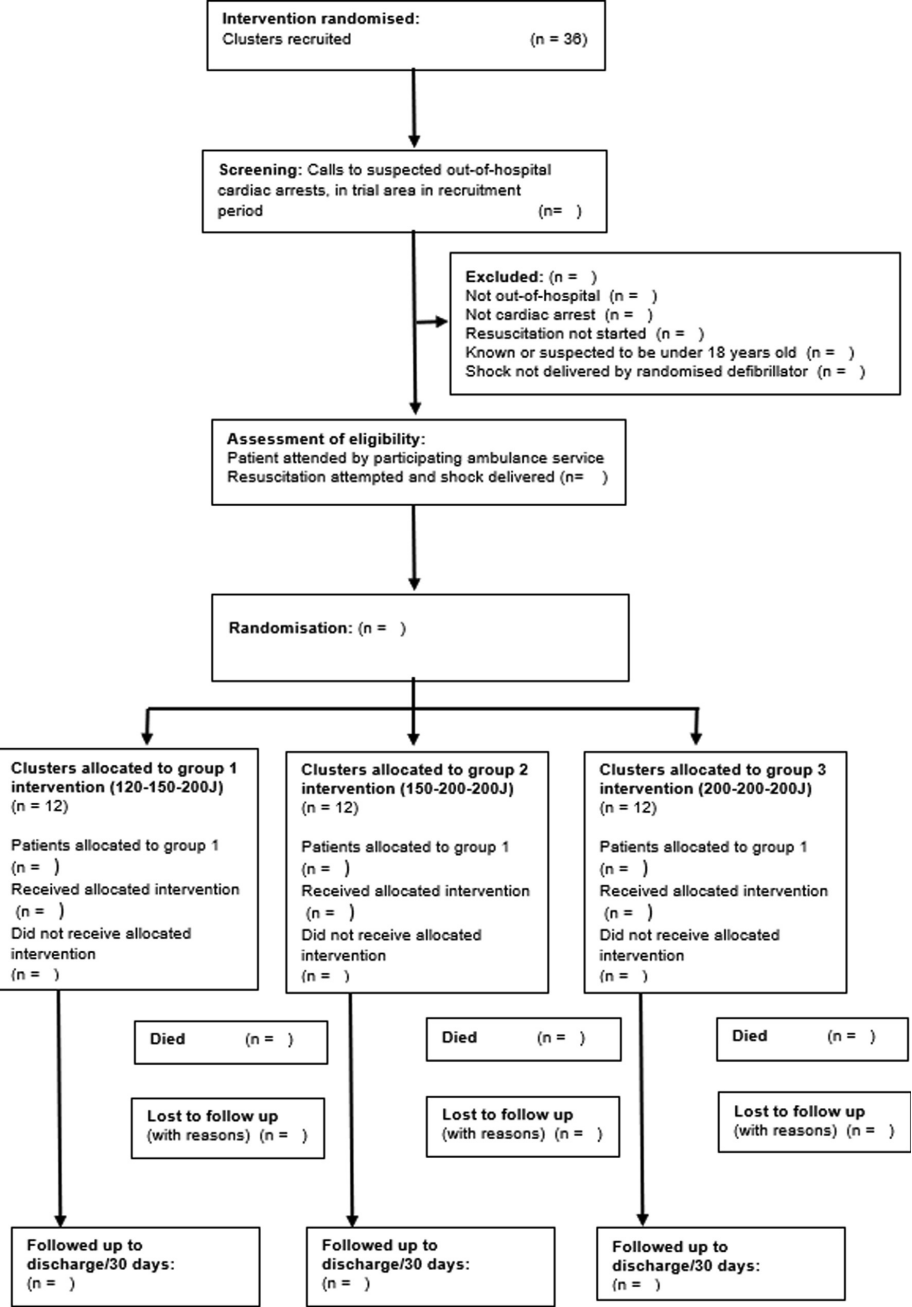
Study CONSORT diagram (POSED).

We will conduct follow-up (assessment of mRS scores) at discharge and at 30 days. Prior to following up patients for whom a consultee declaration was made, we will attempt to ascertain whether the patient has regained capacity. If the patient is thought to have regained capacity, a meeting will be arranged, information provided to the patient and their consent for follow up sought. Fig. 1 shows the study flow diagram.

### Patient & public involvement

Patients and the public are integrally involved in the conception, design, conduct and reporting of POSED. We convened an advisory panel, whom we consulted regarding the rationale and acceptability of the study, consent approach and information provision to survivors and the relatives of non-survivors. The advisory panel were supportive of the study objectives and considered the study would be acceptable to the wider patient and public community.

At the end of the study, we plan to share the study findings with our PPI group who will help us with interpretation for a patient and public perspective. We will jointly construct an information sheet outlining study results. Patient and public representatives who contribute throughout the research will be invited to co-author study outputs including this protocol paper.

### Protocol amendments

We will follow the Health Research Authority (HRA) process to notify sites of any REC-approved protocol amendments. We will also report on the ISRCTN database, trial and funder websites.

### Confidentiality

All data will be stored securely by University of Warwick and held in accordance with Data Protection Act 2018. No personal identifiable data will be held by the study coordinating centre. Personal identifiable data will be shared between the Ambulance service and the receiving hospital to enable follow-up and between the Ambulance service and the patient’s GP to inform the GP of the enrolment and enable follow-up if consent is granted.

### Access to data

An electronic copy of the final study data set shall be sent to the participating ambulance service on completion of the study. On reasonable request to the Chief Investigator, and with appropriate regulatory approvals, study data shall be made available for five years following article publication.

### Dissemination policy

The study team are committed to full disclosure of the results of the trial. Findings will be reported in accordance with CONSORT guidelines,^18^ and we aim to publish in high impact journals. Those who make a significant and sustained contribution to the study will be invited to co-author outputs from this study following ICMJE authorship guidelines. Our patient representatives will assist with dissemination of study results. The funder will take no role in the analysis or interpretation of trial results.

## Discussion

The POSED study aims to demonstrate feasibility of a large-scale trial of shock energy for out-of-hospital cardiac arrest. We aim to test both the performance and acceptability of elements of study design. To identify the best outcome measures we will collect both electrical and clinical outcomes along the patient pathway to recovery. The best outcome measures will be easy and reliable to collect and clinically relevant to patients. For instance, Return Of Organised Rhythm (ROOR), defined as the detection of two QRS complexes < 5 s apart, offers a sensitive marker of shock success but must be assessed post-event by analysis of cardiac rhythm data recorded by defibrillators. It is not known whether timely access to this data is feasible in the UK out-of-hospital setting. Survival, and neurological function at 30 days, although distal clinical markers of shock success, are the preferred outcome measures.^19^ In order to be directly comparable, outcome assessment at the timepoint ‘30 days’ would be preferable to ‘hospital discharge’ since the latter timepoint is liable to between-patient variation. It is not known how easy it will be to access patients for an assessment of neurological function at 30 days since many will have been discharged from hospital at this timepoint.

It is important that cardiac arrest research is acceptable to patients and public. One of the key ethical issues raised by this study is that of consent. In the UK, a condition of seeking consent post-treatment is that the patient or their consultee must be informed and their consent sought at the earliest opportunity.^20^ We will be sensitive to the likely vulnerable emotional state of survivors and relatives in the early stages of their post cardiac-arrest journey. In our experience with the PARAMEDIC2 trial, we found that people were happy to be approached by research paramedics when they had recovered sufficiently to leave the critical care area and been moved to a ward. We consulted with our PPI representatives who felt that this timing is still appropriate.

Sadly, most people who sustain out-of-hospital cardiac arrest do not survive. The study intervention will already have been delivered and if no follow up is conducted, the provision of consent is not necessary. However, the relatives of non-surviving patients may wish to be informed about study participation and a key area of discussion for our PPI representatives was how best to provide this information. Some previous studies conducted in OHCA in the UK, such as the PARAMEDIC, PARAMEDIC2 and AIRWAYS-2 trials, have used a passive approach to informing relatives by placing information in the public domain (e.g., registry offices, ambulance websites).^21^, ^22^, ^23^, ^24^ However, in some studies of cardiac arrest treatment conducted outside the UK it is routine practice to write to the relatives of non-survivors.^25^, ^26^ There was a wide range of opinions expressed focusing around the conflict between the benefits of enhanced transparency from directly informing relatives with the potential for causing distress/harm. Further PPI opinion was sought from members of the University Hospitals Birmingham NHS Foundation Trust Clinical Research Ambassador Group and a Patient Research Ambassador at the Royal Berkshire NHS Foundation Trust. Given that this is a feasibility trial, consensus was reached that the study would assess the feasibility of actively engaging with relatives of non-surviving patients. Our patient and public advisors gave a strong steer away from provision of complex clinical and research information in this first contact. Our advisors helped us to create a letter of condolence that minimises the burden of information whilst empowering people to decide whether and when to seek further information. Opinion was divided regarding the timing of the approach, but we felt that 4–6 weeks post death would be the optimal time, to avoid intrusion on the immediate grieving period. This approach will be monitored throughout the study and amended if it becomes evident that the harms outweigh the benefits.

## Conclusion

The International Liaison Committee on Resuscitation (ILCOR) has highlighted uncertainty about the best energy level with which to deliver shocks for the defibrillation of cardiac arrest. The POSED study is a pragmatic, allocation concealed, open label, cluster randomised controlled study seeking to establish the feasibility of conducting a large-scale trial to address this evidence gap.

## Funding

Helen Pocock, Clinical Doctoral Research Fellow (NIHR-ICA-CDRF-2018-04-ST2-005, is funded by Health Education England (HEE)/National Institute for Health and Care Research (NIHR) for this research project. The views expressed in this publication are those of the author(s) and not necessarily those of the NIHR, (Partner Name), NHS or the UK Department of Health and Social Care. The funder had no input into in study design, collection, analysis or interpretation of data, writing of the report or the decision to submit the article for publication.

GDP is supported by the National Institute for Health Research (NIHR) Applied Research Collaboration (ARC) West Midlands. The views expressed are those of the author(s) and not necessarily those of the NIHR or the Department of Health and Social Care.

## Declaration of interests

HP reports research funding from National Institute for Health and Care Research.

CDD, RL, FM, AC, MAS, PK, AD, DES report no conflicts of interest.

GDP reports research funding from National Institute for Health and Care Research, British Heart Foundation and Resuscitation Council UK. He has volunteer roles with Resuscitation Council UK, European Resuscitation Council and the International Liaison Committee on Resuscitation. He is an editor for the journals Resuscitation and Resuscitation Plus.

## CRediT authorship contribution statement

**Helen Pocock:** Conceptualization, Methodology, Writing – original draft, Funding acquisition. **Charles D. Deakin:** Conceptualization, Methodology, Writing – original draft, Writing – review & editing, Supervision, Funding acquisition. **Ranjit Lall:** Methodology, Writing – review & editing, Supervision, Funding acquisition. **Felix Michelet:** Methodology, Writing – review & editing. **Abraham Contreras:** Methodology, Writing – review & editing. **Mark Ainsworth-Smith:** Methodology, Writing – review & editing. **Phil King:** Methodology, Writing – review & editing. **Anne Devrell:** Methodology, Writing – review & editing. **Debra E. Smith:** Methodology, Writing – review & editing. **Gavin D. Perkins:** Conceptualization, Methodology, Writing – original draft, Writing – review & editing, Supervision, Funding acquisition.

## Figures and Tables

**Figure:**
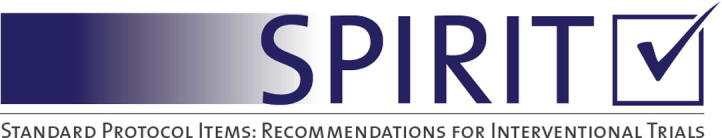


**Table:** 

Section/item	Item No	Description	Addressed on page number
Administrative information			
Title	1	Descriptive title identifying the study design, population, interventions, and, if applicable, trial acronym	Title page
Trial registration	2a	Trial identifier and registry name. If not yet registered, name of intended registry	1
	2b	All items from the World Health Organization Trial Registration Data Set	Appendix A2
Protocol version	3	Date and version identifier	3
Funding	4	Sources and types of financial, material, and other support	15
Roles and responsibilities	5a	Names, affiliations, and roles of protocol contributors	Title page, Credit Author Statement
	5b	Name and contact information for the trial sponsor	3
	5c	Role of study sponsor and funders, if any, in study design; collection, management, analysis, and interpretation of data; writing of the report; and the decision to submit the report for publication, including whether they will have ultimate authority over any of these activities	15
	5d	Composition, roles, and responsibilities of the coordinating centre, steering committee, endpoint adjudication committee, data management team, and other individuals or groups overseeing the trial, if applicable (see Item 21a for data monitoring committee)	10
Introduction			
Background and rationale	6a	Description of research question and justification for undertaking the trial, including summary of relevant studies (published and unpublished) examining benefits and harms for each intervention	2–3
	6b	Explanation for choice of comparators	4
Objectives	7	Specific objectives or hypotheses	3
Trial design	8	Description of trial design including type of trial (eg, parallel group, crossover, factorial, single group), allocation ratio, and framework (eg, superiority, equivalence, noninferiority, exploratory)	4
Methods: Participants, interventions, and outcomes			
Study setting	9	Description of study settings (eg, community clinic, academic hospital) and list of countries where data will be collected. Reference to where list of study sites can be obtained	4
Eligibility criteria	10	Inclusion and exclusion criteria for participants. If applicable, eligibility criteria for study centres and individuals who will perform the interventions (eg, surgeons, psychotherapists)	4
Interventions	11a	Interventions for each group with sufficient detail to allow replication, including how and when they will be administered	4–5
	11b	Criteria for discontinuing or modifying allocated interventions for a given trial participant (eg, drug dose change in response to harms, participant request, or improving/worsening disease)	5
	11c	Strategies to improve adherence to intervention protocols, and any procedures for monitoring adherence (eg, drug tablet return, laboratory tests)	8
	11d	Relevant concomitant care and interventions that are permitted or prohibited during the trial	5
Outcomes	12	Primary, secondary, and other outcomes, including the specific measurement variable (eg, systolic blood pressure), analysis metric (eg, change from baseline, final value, time to event), method of aggregation (eg, median, proportion), and time point for each outcome. Explanation of the clinical relevance of chosen efficacy and harm outcomes is strongly recommended	5–6
Participant timeline	13	Time schedule of enrolment, interventions (including any run-ins and washouts), assessments, and visits for participants. A schematic diagram is highly recommended (see Figure)	Appendix A4
Sample size	14	Estimated number of participants needed to achieve study objectives and how it was determined, including clinical and statistical assumptions supporting any sample size calculations	7
Recruitment	15	Strategies for achieving adequate participant enrolment to reach target sample size	7
Methods: Assignment of interventions (for controlled trials)			
Allocation:			
Sequence generation	16a	Method of generating the allocation sequence (eg, computer-generated random numbers), and list of any factors for stratification. To reduce predictability of a random sequence, details of any planned restriction (eg, blocking) should be provided in a separate document that is unavailable to those who enrol participants or assign interventions	7
Allocation concealment mechanism	16b	Mechanism of implementing the allocation sequence (eg, central telephone; sequentially numbered, opaque, sealed envelopes), describing any steps to conceal the sequence until interventions are assigned	7
Implementation	16c	Who will generate the allocation sequence, who will enrol participants, and who will assign participants to interventions	7
Blinding (masking)	17a	Who will be blinded after assignment to interventions (eg, trial participants, care providers, outcome assessors, data analysts), and how	7
	17b	If blinded, circumstances under which unblinding is permissible, and procedure for revealing a participant’s allocated intervention during the trial	N/A
Methods: Data collection, management, and analysis			
Data collection methods	18a	Plans for assessment and collection of outcome, baseline, and other trial data, including any related processes to promote data quality (eg, duplicate measurements, training of assessors) and a description of study instruments (eg, questionnaires, laboratory tests) along with their reliability and validity, if known. Reference to where data collection forms can be found, if not in the protocol	9
	18b	Plans to promote participant retention and complete follow-up, including list of any outcome data to be collected for participants who discontinue or deviate from intervention protocols	9
Data management	19	Plans for data entry, coding, security, and storage, including any related processes to promote data quality (eg, double data entry; range checks for data values). Reference to where details of data management procedures can be found, if not in the protocol	9
Statistical methods	20a	Statistical methods for analysing primary and secondary outcomes. Reference to where other details of the statistical analysis plan can be found, if not in the protocol	10
	20b	Methods for any additional analyses (eg, subgroup and adjusted analyses)	10
	20c	Definition of analysis population relating to protocol non-adherence (eg, as randomised analysis), and any statistical methods to handle missing data (eg, multiple imputation)	N/A
Methods: Monitoring			
Data monitoring	21a	Composition of data monitoring committee (DMC); summary of its role and reporting structure; statement of whether it is independent from the sponsor and competing interests; and reference to where further details about its charter can be found, if not in the protocol. Alternatively, an explanation of why a DMC is not needed	10
	21b	Description of any interim analyses and stopping guidelines, including who will have access to these interim results and make the final decision to terminate the trial	10
Harms	22	Plans for collecting, assessing, reporting, and managing solicited and spontaneously reported adverse events and other unintended effects of trial interventions or trial conduct	10–11
Auditing	23	Frequency and procedures for auditing trial conduct, if any, and whether the process will be independent from investigators and the sponsor	11
Ethics and dissemination			
Research ethics approval	24	Plans for seeking research ethics committee/institutional review board (REC/IRB) approval	11
Protocol amendments	25	Plans for communicating important protocol modifications (eg, changes to eligibility criteria, outcomes, analyses) to relevant parties (eg, investigators, REC/IRBs, trial participants, trial registries, journals, regulators)	12
Consent or assent	26a	Who will obtain informed consent or assent from potential trial participants or authorised surrogates, and how (see Item 32)	11
	26b	Additional consent provisions for collection and use of participant data and biological specimens in ancillary studies, if applicable	N/A
Confidentiality	27	How personal information about potential and enrolled participants will be collected, shared, and maintained in order to protect confidentiality before, during, and after the trial	12
Declaration of interests	28	Financial and other competing interests for principal investigators for the overall trial and each study site	Declaration of competing interests
Access to data	29	Statement of who will have access to the final trial dataset, and disclosure of contractual agreements that limit such access for investigators	12-13
Ancillary and post-trial care	30	Provisions, if any, for ancillary and post-trial care, and for compensation to those who suffer harm from trial participation	N/A
Dissemination policy	31a	Plans for investigators and sponsor to communicate trial results to participants, healthcare professionals, the public, and other relevant groups (eg, via publication, reporting in results databases, or other data sharing arrangements), including any publication restrictions	13
	31b	Authorship eligibility guidelines and any intended use of professional writers	13
	31c	Plans, if any, for granting public access to the full protocol, participant-level dataset, and statistical code	12–13
Appendices			
Informed consent materials	32	Model consent form and other related documentation given to participants and authorised surrogates	Appendix A5
Biological specimens	33	Plans for collection, laboratory evaluation, and storage of biological specimens for genetic or molecular analysis in the current trial and for future use in ancillary studies, if applicable	N/A

*It is strongly recommended that this checklist be read in conjunction with the SPIRIT 2013 Explanation & Elaboration for important clarification on the items. Amendments to the protocol should be tracked and dated. The SPIRIT checklist is copyrighted by the SPIRIT Group under the Creative Commons “Attribution-NonCommercial-NoDerivs 3.0 Unported” license.

**Table:** 

Data category	Information
Primary registration	ISRCTN16327029, registered 23 June 2021
Funding	NIHR-ICA-2018-04-ST2-005;
Ethics	NHS REC: 20/LO/1242; IRAS no.: 277693
Sponsor	University of Warwick
Title	A feasibility study of Prehospital Optimal Shock Energy for Defibrillation (POSED)
Country of recruitment	UK
Condition of interest	Out-of-hospital cardiac arrest
Interventions	Group 1: 120J (1^st^ shock) + 150J (2^nd^ shock) + 200J (3^rd^ shock) − escalating;
	Group 2: 150J (1^st^ shock) + 200J (2^nd^ shock) + 200J (3^rd^ shock) − escalating;
	Group 3: 200J (1^st^ shock) + 200J (2^nd^ shock) + 200J (3^rd^ shock) − fixed.
Key inclusion and exclusion criteria	Inclusion criteria: Patients sustaining OHCA attended by a crew from participating ambulance service; Resuscitation attempted, and shock delivered as per Resuscitation Council UK and JRCALC guidelines
	Exclusion criteria: Patients known or suspected to be under 18 years old
Study type	Interventional, cluster randomised feasibility study
Date of first enrolment	April 2022
Target sample size	90
Recruitment status	Recruiting to April 2023
Primary outcome	The proportion (%) of eligible patients who are randomised to receive the intervention will be reported.
Secondary outcomes	• Treatment adherence rate. This will be assessed in terms of how many patients received the allocated first shock energy and, where more than one shock was delivered, how many received the correct subsequent shock energies. These will be reported as proportions for each treatment arm and overall.
	• Data completeness of clinical outcomes below:
	o Favourable neurological outcome at 30 days (mRS score)
	o Return Of Organised Rhythm capable of sustaining a pulse (ROOR) 2 min post shock
	o Resulting rhythm (VF/pVT/PEA/asystole) 2 min post shock
	o Re-arrest rate (re-fibrillation)
	o Survived event (return of spontaneous circulation (ROSC) at hospital handover)
	o Survived to hospital discharge
	• Data completeness of process outcomes below:
	o Quality of CPR (chest compression rate, chest compression depth, chest compression fraction, pre-shock pause, post-shock pause)
	o Number of shocks
	o Advanced airway applied (% advanced airway applied and % supraglottic airway or endotracheal tube)
	o Intravenous medicines administered (% cases where medicines administered and % adrenaline, amiodarone)
	o Transported to hospital (% transported)
	These will be reported in terms of the proportion of patients for whom each of these outcomes was collected.

***Key:****CPMS = Central Portfolio Management System; ICA = Integrated Clinical and Practitioner Academic programme; IRAS = Integrated Research Application System; ISRCTN = International Standard Registered Clinical/soCial sTudy Number; J = joules; mRS = modified Rankin scale; NIHR = National Institute for Health and Care Research; OHCA = out-of-hospital cardiac arrest; PEA = pulseless electrical activity; pVT = pulseless ventricular tachycardia; ROOR = return of organised rhythm; ROSC = return of spontaneous circulation; VF = ventricular fibrillation.*

**Figure:**
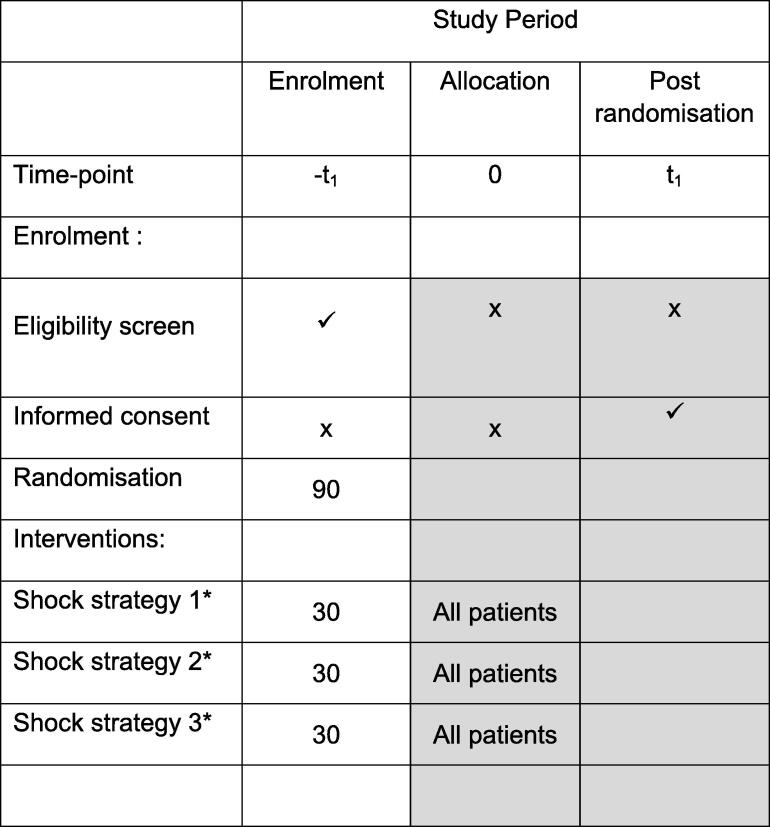


**Table:** 

Group	First shock	Second shock	Subsequent shocks
1	120 J	150 J	200 J
2	150 J	200 J	200 J
3	200 J	200 J	200 J

**Figure:**
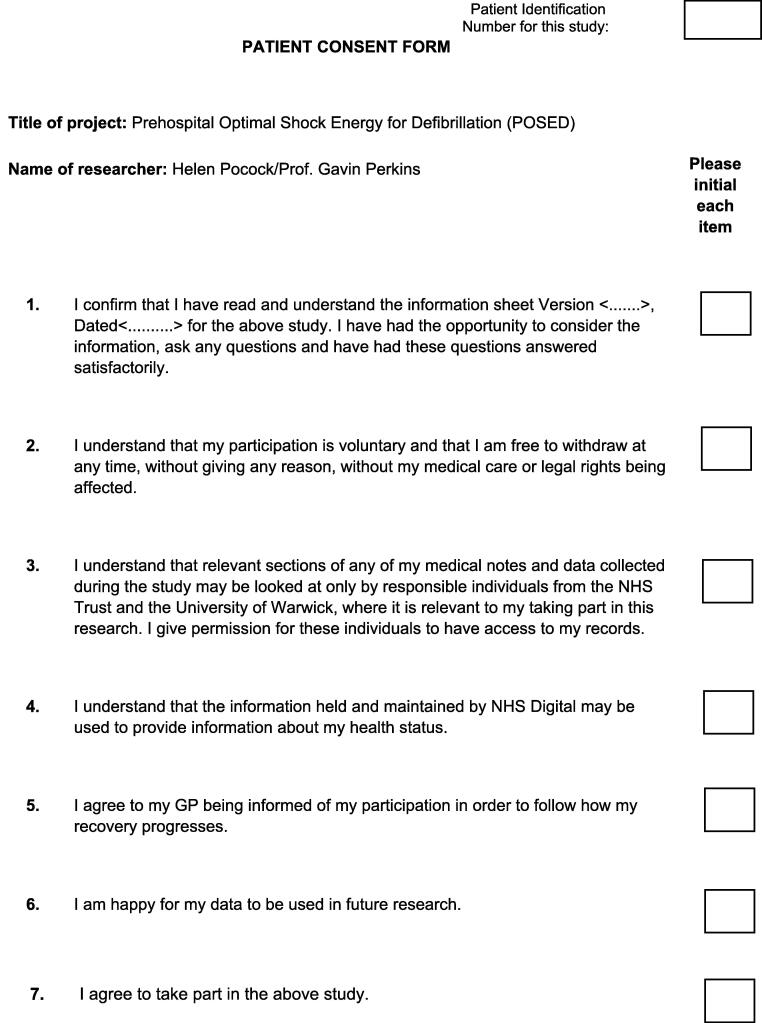


**Figure:**
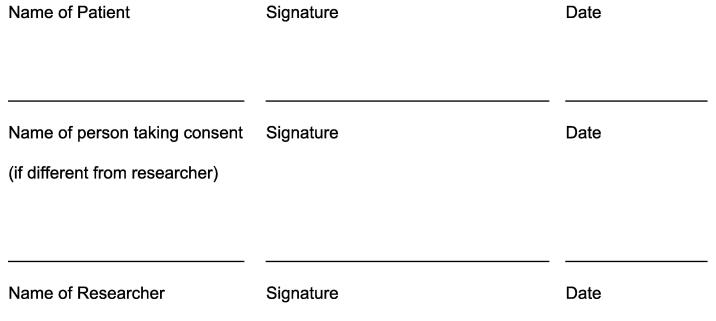


## Appendix A1

SPIRIT 2013 Checklist: Recommended items to address in a clinical trial protocol and related documents*

## Appendix A3 Process outcomes and baseline characteristics

Process outcomes:

o Quality of CPR (chest compression rate, chest compression depth, chest compression fraction, pre-shock pause, post-shock pause)
o Number of shocks
o Advanced airway applied (% advanced airway applied and % supraglottic airway or endotracheal tube)
o Intravenous medicines administered (% cases where medicines administered and % adrenaline, amiodarone)
o Transported to hospital (% transported)

Baseline comparability of the randomised groups:

• Age (median and range)
• Sex (% male)
• Location of arrest (% private residence/public place/other)
• Witnessed vs. unwitnessed event (% witnessed and % bystander/EMS or other healthcare provider/unwitnessed)
• Bystander CPR vs. no bystander CPR (of those not EMS-witnessed, % provided BCPR prior to EMS arrival)
• Type of initial rhythm (% in VF/pVT, PEA or asystole)
• Time from call to application of defibrillator (median and range)
• Aetiology of cardiac arrest (% cardiac vs. non-cardiac cause)

## Introduction

Cardiac arrest, when the heart stops beating, can happen suddenly and unexpectedly. The patient will fall unconscious within seconds, and without treatment, death follows within minutes. We know that the treatments proven to work are chest compressions, known as cardiopulmonary resuscitation (CPR), and electric shocks to the heart, known as defibrillation. The purpose of delivering an electric shock is to kick-start the heart back into beating normally. Electric shock machines, known as defibrillators, can deliver a range of electric shock strengths (low, medium or high). But we don’t know the best energy level for these shocks. Too little energy may fail to defibrillate the heart whilst too much energy may damage it. Since the best energy level is not known, the UK Ambulance Services give different shock strengths (low, medium or high) depending on local protocols.

When the effects of different treatments are not known, researchers may conduct a clinical trial to find out. The three shock strategies being compared in this study represent standard of care in current use in UK Ambulance Services. The three arms are as follows:Trials of this type are usually large, so it is necessary to first find out whether these trials are possible and anticipate any problems. Conducting a ‘feasibility study’ helps to ensure that time and money are well spent on a full-scale trial*.* This study involves a single UK ambulance service and we will enrol 90 patients to assess whether it would be feasible to conduct a full clinical trial.

## This feasibility study will help to design a trial to determine the best electric shock strength for defibrillation

### How am I involved in the feasibility study?

Immediately following your cardiac arrest you were shocked using a defibrillator, the energy level (low, medium or high) having being randomly allocated to the defibrillator (a computer allocated your treatment by chance, which is like making a choice by tossing a coin). This is important as it ensures the treatment groups are as similar as possible so that any difference in outcomes are due only to the treatment received. The paramedics who treated you will know which treatment you received but neither you nor the medical team in hospital will be told. This is to ensure that decisions regarding subsequent treatments cannot be influenced.

When your heart stopped you lost consciousness immediately. As a result, the Paramedics were unable to discuss with you your views about participation in the study at the time of your cardiac arrest. It was also not possible for the Paramedics to speak to those close to you to obtain consent as this may have delayed your emergency treatment. In this situation the Ambulance Service are authorised to apply deferred consent at the time of the emergency.

## We are now asking your permission to continue onto the next phase of the study

### What will happen to me if I agree to continue to take part?

You have already received the treatment that forms part of this study and data regarding your cardiac arrest has been recorded. No further study related treatments are required.

We now wish to follow how your recovery progresses up to 30 days following your cardiac arrest. We proposed to do this in two ways:

• We will visit you and ask you to sign a copy of the consent form. We will then complete a short assessment of how well you are recovering. We propose to do this by discussing your progress with you and, if you choose, your relative/friend. This will take place during a single visit, lasting around 20 minutes.
• We will ask the doctors/nurses/paramedics to look at your NHS records to let us
• know how you are getting on. This will include information about how long you spent in hospital, what treatments you required and how well you recovered. Some of this information will be obtained from NHS Digital who look after health and care information.

### Do I have to take part?

You do not have to agree to take part. If, as is your right, you choose not to participate in any aspect of the study any further, it will not affect the treatment or care that you receive in any way either now or in the future. You can indicate if you would like to take part in each aspect of the follow up on the consent form which will need to be signed and dated. If you initially consent to take part in the study you still have the right to withdraw from the research later if you wish.

### What are the possible risks and benefits of taking part?

The risks from you continuing in the trial are small. Although most people remember very little about their cardiac arrest, receiving a visit from a researcher could be upsetting. Our trained research staff can talk to you about any such feelings and can offer to put you in contact with professional services who can help if required. Some people have found it beneficial to receive a visit from a researcher as they have been able to have their questions answered about the treatment they received. Continuing to be part of the trial will require a modest time commitment for this visit. However, the information we collect from you will help us to develop a trial to understand know how best to treat people with electric shocks as a treatment for cardiac arrest. This valuable information could shape medical care across the world, potentially saving more lives and producing better long term outcomes.

### How will we use information about you?

The University of Warwick is the sponsor for this study based in the United Kingdom and will act as the data controller for this study. This means that we are responsible for looking after your information and using it properly. South Central Ambulance Service NHS Foundation Trust (SCAS) will collect information from you and your medical records for this research study in accordance with our instructions.

SCAS will keep your name, NHS number and contact details confidential and will not pass this information to the University of Warwick. SCAS will use this information as needed, to contact you and/or your GP about the research study, and make sure that relevant information about the study is recorded for your care, and to oversee the quality of the study. This data will be retained until submission of the final research report to the funder. Your GP may be asked to provide medical record information unless you decline consent for this.

The people who analyse the information will not be able to identify you and will not be able to find out your name, NHS number or contact details.

The University of Warwick will retain study data for a minimum of 10 years after the study has finished.

Your rights to access, change or move your information are limited, as we need to manage your information in specific ways in order for the research to be reliable and accurate. If you withdraw from the study, we will keep the information about you that we have already obtained. To safeguard your rights, we will use the minimum personally-identifiable information possible.

Individuals from the University of Warwick and regulatory organisations may look at your medical and research records to check the accuracy of the research study.

You can find out more about how we use your information at https://warwick.ac.uk/services/idc/dataprotection/privacynotices/researchprivacynotice.

People will use this information to do the research or to check your records to make sure that the research is being done properly. We will keep all information about you safe and secure. People who do not need to know who you are will not be able to see your name or contact details. The University of Warwick will not hold information that will enable us to identify you by name. Your data will have a code number instead. Once we have finished the study, we will keep some of the data so we can check the results. We will write our reports in a way that no-one can work out that you took part in the study.

When you agree to take part in a research study, the information about your health and care may be provided to researchers running other research studies in this organisation and in other organisations. These organisations may be universities, NHS organisations or companies involved in health and care research in this country or abroad. Your information will only be used by organisations and researchers to conduct research in accordance with the UK Policy Framework for Health and Social Care Research**.** This information will not identify you and will not be combined with other information in a way that could identify you. The information will only be used for the purpose of health and care research, and cannot be used to contact you or to affect your care. It will not be used to make decisions about future services available to you, such as insurance.

### What are your choices about how your information is used?

You can stop being part of the study at any time, without giving a reason, but we will keep information about you that we already have.

We need to manage your records in specific ways for the research to be reliable. This means that we won’t be able to let you see or change the data we hold about you.

### Where can you find out more about how your information is used?

You can find out more about how we use your information:

• at www.hra.nhs.uk/information-about-patients/
• by asking one of the research team
• by sending an email to [email], or
• by ringing us on [phone number].

### What will happen to the results of the research study?

The study is expected to take around three years. We will endeavour to make sure the results of the study are shared widely. We will share the results with other healthcare professionals and will publish in medical journals. When any information from the study is published it will not contain personal information, and it will not be possible to identify you. After the trial has finished we will not be informing participants which treatment they received but if you would like a copy of the published results, please contact the Study Team (contact details below).

### How have patients and public been involved in the study?

We have established a patient and public advisory panel for this study. They have agreed that the study is important and feel that, since the shock strength you would receive were you not included in the study would be determined by which ambulance service treats you, individuals will not be disadvantaged by being included in the research. Their opinions have shaped our thinking, especially around the issue of informing people about their inclusion in the study. Their suggestions have been incorporated into the design of the study particularly the acceptability and readability of the study documents.

### Who has reviewed the study?

All research in the NHS is reviewed by an independent group of people, called a Research Ethics Committee, to protect your safety, rights, wellbeing and dignity. This study was reviewed and given a favourable opinion by the <XXXXX> Research Ethics Committee on <DATE>. The study has also been reviewed by the National Institute for Healthcare Research (NIHR) Integrated Clinical Academic programme panel.

### What happens next?

You do not have to do anything now. A member of the Research Team will contact you again to discuss this further.

### Who do I contact for more information?

(1) PRINCIPAL INVESTIGATOR FOR LOCAL SITE NAME, [email].
(2) RESEARCH PARAMEDIC NAME, [email].
(3) Trial Team at the University of Warwick: [email]. Tel: [mobile].

### Who should I contact if I wish to make a complaint?

Any complaint about the way you have been dealt with during the study or any possible harm you might have suffered will be addressed. Please address your complaint to the person below, who is a senior University of Warwick official entirely independent of this study:

Head of Research Governance

Research & Impact Services, University House, University of Warwick, Coventry

CV4 8UW. Email: researchgovernance@warwick.ac.uk; Tel: 024 76 522746

If you wish to raise a complaint on how we have handled your personal data, you can contact the University of Warwick’s Data Protection Officer, who will investigate the matter: DPO@warwick.ac.uk.

If you are not satisfied with our response or believe we are processing your personal data in a way that is not lawful you can complain to the Information Commissioner’s Office (ICO).
